# Single-Case Experimental Evidence on Social Problem-Solving Interventions for Individuals with Autism: A Three-Level Meta-Analysis

**DOI:** 10.3390/bs16071129

**Published:** 2026-07-06

**Authors:** Shaoju Jin, Chunyan Zhou, Huan Huang, Xiaolong Zhou

**Affiliations:** 1 Faculty of Education, East China Normal University, Shanghai 200062, China; 52274109003@stu.ecnu.edu.cn (S.J.); 52284109002@stu.ecnu.edu.cn (H.H.); 2 Sichuan Revolutionary Old Area Development Research Center, Sichuan University of Arts and Science, Dazhou 635000, China; 20180040.z-cy_@sasu.edu.cn

**Keywords:** autism spectrum disorder, social problem-solving, single-case experimental design, three-level meta-analysis

## Abstract

Social problem-solving (SPS) difficulties may affect social, communicative, and adaptive functioning among individuals with autism. This study conducted a three-level meta-analysis of SPS interventions in single-case experimental design (SCED) research. Twenty-one SCED studies published between 2002 and 2025 met the inclusion criteria, yielding 114 dependent-variable-level effect sizes nested within 59 participant clusters. The unconditional three-level model showed a positive and statistically significant pooled effect, 1.741 (*SE* = 0.087, 95% *CI* [1.570, 1.911], *p* < 0.001), with low approximate total heterogeneity (*I*^2^ = 24.48%). Moderator analyses did not identify clear statistically significant predictors of effect-size variability across SPS centrality, intervention setting, intervention type, implementer type, participant characteristics, or methodological quality. Sensitivity and study-level publication-bias analyses provided no clear indication that the findings were driven by a single influential study or by obvious small-study effects. Overall, targeted outcomes improved from baseline to intervention phases in studies of SPS-related intervention packages. Because many studies used multi-component intervention packages, the observed effects reflect the combined impact of interventions in which SPS-related components were embedded, and it is not possible to isolate the independent contribution of SPS components based on the available SCED evidence.

## 1. Introduction

Autism spectrum disorder (ASD) is characterized by persistent difficulties in social communication, interpersonal interaction, and adaptive social functioning, which can affect peer relationships, academic participation, community engagement, and long-term psychosocial adjustment (American Psychiatric Association, 2013; Bauminger & Kasari, 2000). Although many individuals with autism acquire discrete social behaviors through intervention, they may continue to experience difficulty applying these behaviors flexibly across dynamic social contexts. Therefore, increasing attention has been directed toward interventions that target not only observable social behaviors but also the problem-solving processes that support adaptive responding in everyday situations.

Social problem solving (SPS) is an important intervention target within social–emotional learning because it involves identifying social or functionally meaningful problems, interpreting contextual cues, generating possible responses, evaluating consequences, and selecting or implementing adaptive solutions (CASEL, 2018; Durlak et al., 2011). SPS differs from broader social skills instruction, which may focus primarily on discrete behaviors such as greetings, conversational turn-taking, or eye contact. This distinction is important for autism intervention research because SPS emphasizes social reasoning, emotion regulation, executive functioning, cognitive flexibility, and adaptive decision making, all of which may be relevant to the social and functional challenges experienced by individuals with autism (Hill, 2004; White et al., 2014; McNair et al., 2024).

The theoretical foundation of SPS is closely related to the Social Information Processing model, which conceptualizes social behavior as a sequence of cognitive processes involving cue encoding, cue interpretation, goal clarification, response generation, response evaluation, and behavioral enactment (Crick & Dodge, 1994). Later extensions emphasized the role of affective processes in social decision making (Lemerise & Arsenio, 2000). Related problem-solving frameworks also emphasize explicit cognitive mediation and metacognitive strategy use in adaptive behavior (Nezu, 2004; Shure & Spivack, 1982). Together, these perspectives support the view that SPS can be taught as a structured cognitive–behavioral process rather than treated only as a general outcome of social skills training.

Empirical support for social and social–cognitive interventions for individuals with autism has grown over the past two decades. Prior syntheses have generally reported positive effects of social skills and related interventions (Bellini et al., 2007; Gates et al., 2017), and recent group-comparison evidence has also suggested positive effects of SPS interventions on SPS competence and related social–emotional outcomes (Jin et al., 2025). However, the SPS evidence base remains conceptually heterogeneous. Some studies teach SPS as a comprehensive instructional sequence, whereas others target functional problem solving or embed selected SPS components within broader intervention packages. Several single-case experimental designs (SCED) studies have examined SPS-related interventions for individuals with autism and reported improvements in targeted social, communicative, functional, or adaptive outcomes (Dotto-Fojut et al., 2011; Lora et al., 2020; Suarez et al., 2022; Yakubova & Zeleke, 2016). Because these studies vary in intervention components, participant characteristics, outcome measures, and the extent to which SPS is the central instructional target, a quantitative synthesis of SCED evidence is needed to estimate the overall baseline-to-intervention effect and examine whether study, participant, or intervention characteristics are associated with effect-size variability.

SCEDs are frequently used in autism intervention research because they are well suited for evaluating individualized and context-sensitive interventions (Reichow & Volkmar, 2010; Wang et al., 2013). SCED studies allow researchers to examine within-participant change, repeatedly measure behavior across baseline and intervention phases, and evaluate intervention effects in applied contexts (Dotto-Fojut et al., 2011; Lora et al., 2020; Suarez et al., 2022; Yakubova & Zeleke, 2016). These features are particularly relevant for SPS interventions, which often involve individualized goals, repeated practice, instructional prompts, performance feedback, and adaptation to specific social or functional problem situations.

At the same time, synthesizing SCED evidence presents important statistical challenges. SCED studies often generate multiple effect sizes from different dependent variables, participants, or contexts within the same study, which violates the assumption of statistical independence used in many conventional meta-analytic approaches. Earlier SCED syntheses often relied on visual analysis or nonoverlap indices, which can provide useful descriptive information but may offer limited precision for estimating pooled effects and heterogeneity (Brossart et al., 2006). Three-level meta-analytic models provide a useful framework for addressing this dependency by modeling effect sizes at the dependent-variable level, participant-cluster level, and study level simultaneously (Cheung, 2013; Van den Noortgate et al., 2013). This approach allows researchers to retain multiple effect sizes from the same study while accounting for dependency among effect-size estimates.

Building on prior SCED meta-analytic work in autism intervention research (Wang et al., 2013), the present study synthesized SCED evidence on SPS interventions for individuals with autism using three-level meta-analytic models. The study aimed to: (1) estimate the overall baseline-to-intervention effect of interventions incorporating SPS-related instruction; and (2) explore whether SPS centrality, participant characteristics, intervention features, setting, implementer type, and methodological quality were associated with effect-size variability. By clarifying the role of SPS-related instruction and accounting for dependency among SCED effect sizes, this review provides a more precise synthesis of the current evidence base and identifies directions for future SPS intervention research.

## 2. Methods

This systematic review with a three-level meta-analysis was conducted and reported in accordance with the Preferred Reporting Items for Systematic Reviews and Meta-Analyses (PRISMA 2020) statement (see Supplementary Table S2; Page et al., 2021). The study protocol was preregistered on the Open Science Framework prior to data extraction (OSF: https://osf.io/zex2q, accessed on 9 May 2026).

### 2.1. Search Strategy

We systematically searched six electronic databases: Web of Science, APA PsycINFO, SpringerLink, ERIC, PubMed, and Scopus. Searches included studies published between January 2000 and December 2025. The final database search was completed on 6 January 2026. Search terms were applied to titles, abstracts, and keywords and covered three domains: (1) study design (e.g., single-case experimental design, single-subject, SCED); (2) population (e.g., autism spectrum disorder, ASD, autism, Asperger); and (3) intervention focus (e.g., social problem-solving, problem-solving skills, social decision-making, interpersonal problem solving). Terms within each domain were combined with “OR,” and the three domains were linked with “AND.” Search syntax was adapted to each database’s indexing conventions (see Supplementary Table S1). Reference lists of all included studies and relevant systematic reviews were additionally screened to identify eligible records not captured by the database search. Only peer-reviewed journal articles published in English were considered for inclusion.

### 2.2. Operational Definition and Eligibility Criteria

In this review, SPS was operationalized as a taught process through which individuals with autism identify and respond adaptively to socially situated or functionally meaningful problems, consistent with social problem-solving and social information-processing frameworks (Crick & Dodge, 1994; Nezu, 2004; D’Zurilla et al., 2012). SPS components included problem identification, cue interpretation, response generation or selection, evaluation of consequences or appropriateness, implementation of an adaptive response, self-questioning or self-monitoring, and transfer or generalization across contexts. Studies were included only when problem solving was an explicit instructional target or when SPS processes were identifiable as core intervention components. A problem context alone was not sufficient for inclusion; interventions focused solely on general social skills, adaptive behavior, academic instruction, task completion, or fixed behavioral routines without an identifiable SPS process were excluded.

Studies were eligible if they met all of the following criteria: (1) participants had a formal diagnosis of ASD, including autism, Asperger syndrome, or pervasive developmental disorder-not otherwise specified (PDD-NOS), based on DSM-IV, DSM-5, or ICD-10/11 criteria, or received services under an autism eligibility category; (2) the study employed a single-case experimental design, such as multiple baseline, multiple probe, alternating treatment, reversal/withdrawal, or changing criterion designs; (3) the intervention explicitly taught an SPS routine or one or more observable SPS components; (4) the target context involved a socially situated or functionally meaningful problem or barrier; (5) the study reported sufficient quantitative data across baseline and intervention phases to compute or estimate an effect size; and (6) the study received a quality rating of 2 or above on the What Works Clearinghouse (WWC) single-case design standards.

Studies were excluded if they: (1) were dissertations, theses, conference papers, or other forms of grey literature; (2) employed group-based experimental designs rather than SCEDs; (3) included participants without a confirmed ASD diagnosis and did not report autistic participant data separately; (4) focused exclusively on general social skills instruction, adaptive behavior, academic instruction, or task completion without an identifiable SPS component; (5) lacked sufficient data for effect-size calculation after attempts to contact authors; or (6) failed to meet WWC methodological standards (score = 1). For mixed-sample studies, only data from individuals with autism were retained when these data were separately extractable.

### 2.3. Data Extraction and Coding

Two reviewers independently extracted study, participant, methodological, and outcome data using a standardized coding form. When studies reported multiple participants, outcomes, targets, or settings, each eligible DV-level series was coded as a separate effect-size estimate. Effect sizes were nested within participant clusters, which were nested within studies. When numerical data were unavailable, values were digitized from graphs using WebPlotDigitizer (Version 4.5). To assess digitization reliability, a second coder independently digitized a randomly selected 30% of the graphical data, yielding 98% agreement between coders; any discrepancies were resolved through recalibration and consensus discussion. Only baseline and intervention phases were included in the primary analyses; maintenance, follow-up, and generalization data were coded descriptively when reported.

To address construct heterogeneity, each study was coded for SPS centrality, defined as the extent to which problem solving was the primary instructional target or an embedded component of a broader intervention. Three categories were used: comprehensive SPS, functional problem-solving, and embedded SPS-component interventions. Comprehensive SPS interventions taught a relatively complete, multi-step SPS process. Functional problem-solving interventions taught problem resolution in more constrained, function-based contexts. Embedded SPS-component interventions included SPS elements within broader communication, vocational, or functional communication interventions. Supplementary Table S4 provides the SPS component coding and centrality classification for each included study.

Inter-rater reliability was assessed at three stages: title and abstract screening, full-text eligibility evaluation, and data extraction. Agreement was calculated using percentage agreement and Cohen’s κ. Results indicated substantial to almost perfect agreement at all three stages (κ range = 0.83–0.91), consistent with established benchmarks (Landis & Koch, 1977). All discrepancies were resolved through discussion or consultation with a third reviewer.

### 2.4. Risk of Bias Assessment

Methodological quality was evaluated using the WWC Standards for Single-Case Design Research (What Works Clearinghouse, 2020), which are widely used to assess the methodological rigor of SCED research. The WWC framework evaluates studies across three domains: (1) design and implementation, including documentation of phase changes and demonstration of experimental control; (2) data collection and measurement, including operational definitions, fidelity of measurement, and adequacy of data points; and (3) internal validity, including replication of intervention effects across phases, participants, or settings. Each study was assigned one of three ratings: Meets WWC Standards Without Reservations (score = 3), Meets WWC Standards With Reservations (score = 2), or Does Not Meet WWC Standards (score = 1). Two reviewers independently rated each study; discrepancies were resolved through consensus discussion or third-reviewer adjudication. Only studies scoring 2 or higher were retained for quantitative synthesis. Individual WWC ratings are reported in Supplementary Table S3.

### 2.5. Statistical Analysis

All analyses were conducted in R (Version 4.5.0) using the *metafor* package (Version 5.0.1) (Viechtbauer, 2010). Three-level random-effects meta-analytic models were fitted to synthesize dependent effect sizes derived from SCED data. This approach was used because multiple DV-level effect sizes were nested within participant clusters, which were nested within studies (Cheung, 2013; Van den Noortgate et al., 2013).

The primary effect size metric was a standardized mean difference (SMD) derived from procedures commonly used in single-case meta-analytic research (Dowdy et al., 2021; Hedges et al., 2012; Wang et al., 2013). Prior to effect size computation, raw data from outcome graphs in each included study were digitized using WebPlotDigitizer. For each dependent variable (DV), baseline and intervention data points were standardized using the combined mean and standard deviation across phases. Standardized scores were then shifted so that the baseline phase mean equaled zero. Under this procedure, the mean standardized score during the intervention phase represented the effect size estimate for that DV. For outcomes in which lower scores reflected improvement, data were reversed before analysis so that positive values consistently indicated improvement in SPS or SPS-related performance. Effect sizes were interpreted descriptively as standardized baseline-to-intervention phase changes, with larger positive values indicating greater improvement relative to baseline.

The model partitioned variance across three levels: dependent variables (Level 1), participant clusters (Level 2), and studies (Level 3). The unconditional three-level model was specified as follows:

Level 1 (DV level): *Y_ijk_* = *π*_0*jk*_ + *e_ijk_*;

Level 2 (Participant cluster level): *π*_0*jk*_ = *β*_00*k*_ + *r*_0*jk*_;

Level 3 (Study level): *β*_00*k*_ = *γ*_000_ + *u*_00*k*_/

Where *Y_ijk_* represents the effect size estimate for DV *i*, participant *j*, and study *k*; *γ*_000_ represents the overall pooled effect; and *e_ijk_*, *r*_0*jk*_, and *u*_00*k*_ are residual error terms at Levels 1–3, respectively. Level 1 represents individual effect size estimates (DVs); Level 2 captures variance among participants within a study; and Level 3 captures variance among studies. Parameters were estimated using restricted maximum likelihood (REML). Heterogeneity was partitioned into between-study (*τ*^3^_2_) and within-study between-participant (*τ*^2^_2_) components, with the proportion of variance at each level quantified using the *I*^2^ statistic. Values of 25%, 50%, and 75% were interpreted as indicative of low, moderate, and high heterogeneity, respectively (Higgins et al., 2003).

Moderator analyses were conducted using separate three-level meta-analytic models. SPS centrality was included as a construct-validity moderator to examine whether effects varied according to whether SPS was the central intervention focus or an embedded component within a broader intervention package. Additional moderators included intervention setting, intervention type, implementer type, participant age, participant gender composition, and WWC methodological quality rating. Moderators were analyzed separately to reduce multicollinearity and improve interpretability. Omnibus moderator effects were evaluated using Wald-type QM tests. Given the modest number of included studies and small moderator categories, moderator findings were interpreted as exploratory.

The robustness of pooled estimates was evaluated through leave-one-out sensitivity analyses, in which each study was sequentially removed to assess its influence on the overall estimate. Publication bias was examined via funnel plot inspection, Egger’s regression test (Egger et al., 1997), and Rosenthal’s fail-safe N (Rosenthal, 1979). Where asymmetry was detected, the trim-and-fill procedure was applied to impute potentially missing studies and generate bias-adjusted estimates (Duval & Tweedie, 2000). Because conventional publication-bias diagnostics assume independent effect sizes, these analyses were conducted at the study level and interpreted cautiously (Dowdy et al., 2021; Gage et al., 2017; Tincani & Travers, 2019).

## 3. Results

### 3.1. Study Selection

**Figure 1:**
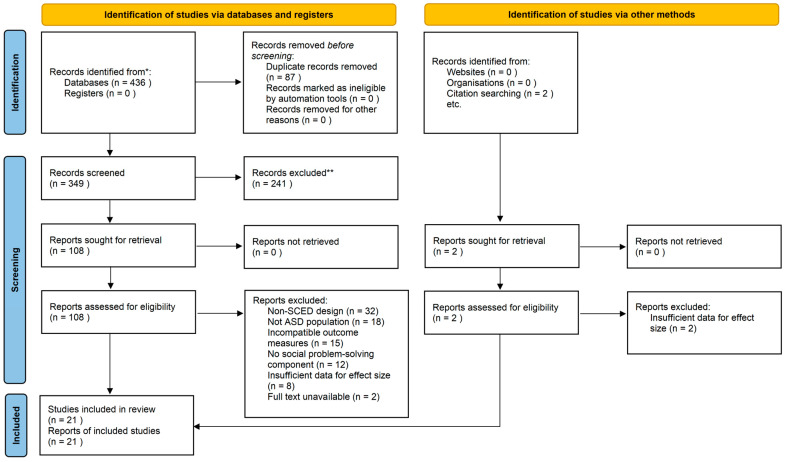
The PRISMA 2020 flow diagram of study identification, screening, eligibility, and inclusion. **Note.** * Records identified from database searches. ** Records excluded after title and abstract screening because they did not meet the inclusion criteria.

The initial database search identified 436 records (Figure 1). After removal of 87 duplicate records, 349 records were screened based on titles and abstracts. Two independent reviewers (S.J. and C.Z.) screened all records, excluding 241 records during initial screening. The remaining 108 reports were retrieved for full-text assessment. Of these, 87 reports were excluded for the following reasons: non-SCED (n = 32), participants without a confirmed ASD diagnosis (n = 18), incompatible outcome measures (n = 15), absence of an explicit SPS or SPS-component intervention (n = 12), insufficient data to compute effect sizes (n = 8), and full text unavailable (n = 2). In addition, two records were identified through manual screening of reference lists and citation searching. However, neither record met the final eligibility criteria for quantitative synthesis. After application of the WWC single-case design standards and verification of data completeness, 21 studies were retained for the final meta-analysis (see Supplementary Materials S1). Disagreements during screening and eligibility evaluation were resolved through discussion, and unresolved disagreements were adjudicated by a third reviewer (H.H.).

### 3.2. Characteristics of Included Studies

The final analytic sample comprised 21 SCED studies published between 2002 and 2025. The final analytic dataset included 114 DV-level effect sizes nested within 59 autistic participant clusters. Participants ranged in age from 4 to 25 years (M = 14.47 years), and most participant clusters were male (54 males, 5 females; 91.5% male). Studies were conducted primarily in the United States, with additional studies from Canada, Australia, and Jordan. Interventions were implemented across general education classrooms, special education settings, clinics, community environments, vocational or transition-related settings, and home-based programs. Implementers included researchers, teachers, therapists, paraprofessionals, and caregivers.

All included studies employed SCED methodologies, most commonly multiple-baseline or multiple-probe designs. To address construct heterogeneity, studies were classified into three SPS centrality categories: Comprehensive SPS interventions, Functional problem-solving interventions, and Embedded SPS-component interventions. Six studies were coded as Comprehensive SPS interventions, seven as Functional problem-solving interventions, and eight as Embedded SPS-component interventions.

### 3.3. Risk of Bias Within Studies

Fifteen studies (71.4%) received a rating of Meets WWC Standards Without Reservations (score = 3), indicating relatively strong methodological rigor in terms of phase documentation, operational definitions of dependent variables, and demonstration of experimental control. The remaining six studies (28.6%) received a rating of Meets WWC Standards With Reservations (score = 2), primarily because of insufficient data points in one or more phases or limited replication of effects across conditions. No study received a score of 1 (Does Not Meet Standards), indicating that all studies in the final analytic sample met the minimum WWC threshold for inclusion in the quantitative synthesis. Individual study ratings are presented in Supplementary Table S3.

### 3.4. Overall Effect Size

**Figure 2:**
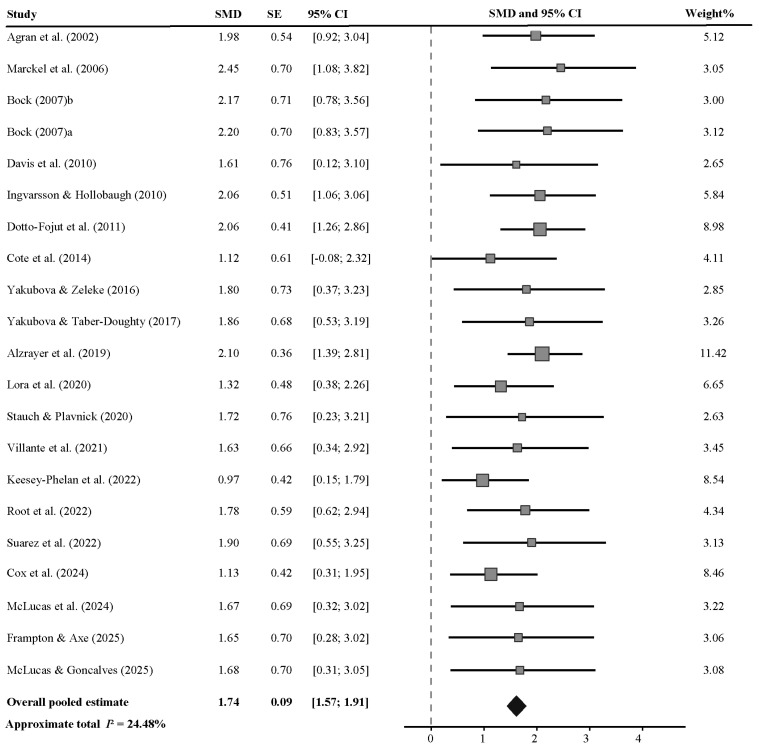
Forest plot of study-level effect sizes for SPS and SPS-component intervention for individuals with autism. Squares represent study-level standardized mean differences, and horizontal lines indicate 95% confidence intervals. Study-level estimates are shown for visualization; the pooled estimate was obtained from the three-level meta-analytic model using all DV-level effect sizes. The diamond represents the pooled estimate. Positive values indicate improvement relative to baseline.

The unconditional three-level meta-analytic model estimated a positive and statistically significant pooled effect of SPS and SPS-component intervention packages on SPS-related outcomes, with a pooled estimate of 1.741 (*SE* = 0.087, 95% *CI* [1.570, 1.911], *p* < 0.001; see Figure 2). This estimate indicates that, on average, SPS-related outcomes improved from baseline to intervention phases. Because many included studies evaluated multi-component intervention packages, the pooled estimate should be interpreted as the effect of packages containing SPS or SPS-embedded components rather than as the isolated effect of SPS components alone.

### 3.5. Variance Components and Heterogeneity

The unconditional three-level meta-analytic model estimated a between-study variance component of 0.0749 and a within-study between-participant variance component of 0.0227. The approximate total *I*^2^ was 24.48%, suggesting low heterogeneity in the overall model. These results indicate that some variability remained across studies and participant clusters, although the overall degree of heterogeneity was limited. Descriptive effect-size distributions and variance component estimates are presented in Table 1.

### 3.6. Moderator Analyses

Moderator analyses were conducted using separate three-level meta-analytic models with DV-level effect sizes nested within participant clusters and studies. SPS centrality was examined as a construct-validity moderator, and additional moderators included intervention setting, WWC methodological quality rating, implementer type, intervention type, participant age, and participant gender composition. Omnibus moderator effects were evaluated using Wald-type QM tests.

SPS centrality did not significantly moderate effect sizes, QM(2) = 0.58, p = 0.747. Intervention setting also did not reach statistical significance, QM(2) = 5.55, p = 0.062, although estimates were numerically higher for school-based and home-based settings than for clinical/community/transition settings. WWC methodological quality rating was at the conventional significance threshold, QM(1) = 3.85, p = 0.050, and was interpreted cautiously.

No clear moderation effects were found for implementer type, QM(2) = 0.91, p = 0.635; intervention type, QM(5) = 2.99, p = 0.701; participant age, QM(1) = 0.27, p = 0.606; or participant gender composition, QM(1) = 0.08, p = 0.773. Residual between-study variance and proportional changes in *τ*^2^_3_ are reported descriptively in Table 2. Given the modest number of included studies and small cell sizes in some categories, these moderator results should be interpreted cautiously.

### 3.7. Sensitivity Analysis and Publication Bias

**Figure 3:**
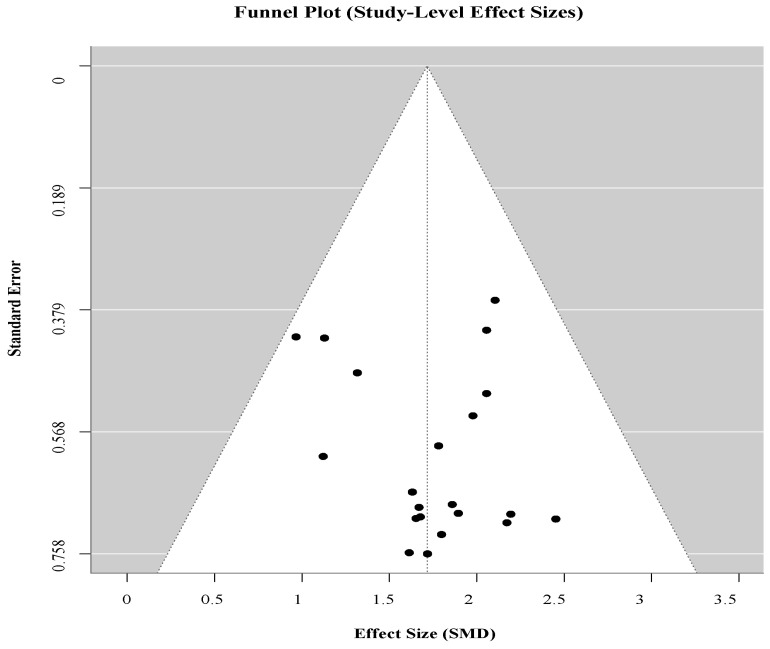
Funnel plot of study-level effect sizes for assessing potential publication bias. Each point represents a study-level standardized mean difference.

A leave-one-out sensitivity analysis was conducted to evaluate the robustness of the pooled estimate. Across all iterations, the pooled estimates remained positive and statistically significant, ranging from 1.712 to 1.826, indicating that no single study exerted disproportionate influence on the results. Publication bias was examined at the study level using funnel plot inspection, Egger’s regression test, Rosenthal’s fail-safe N, and trim-and-fill procedures. The funnel plot showed an approximately symmetrical distribution of study-level effect sizes around the pooled estimate, although a slight clustering of larger positive effects was observed (Figure 3). Egger’s regression test was not statistically significant, z = 0.34, *p* = 0.737, suggesting no clear evidence of small-study effects. Rosenthal’s fail-safe N was 1458, indicating that a large number of unpublished null-effect studies would be required to reduce the overall effect to non-significance. The trim-and-fill procedure imputed no missing studies, and the adjusted estimate was therefore unchanged.

Overall, the sensitivity and publication-bias analyses provided no robust or definitive evidence that the main findings were driven by a single influential study or by small-study effects. However, these results should be interpreted cautiously, as conventional publication-bias methods have limited sensitivity in single-case experimental design meta-analyses, particularly given the small number of independent studies and the dependency among effect sizes.

## 4. Discussion

### 4.1. Overall Effectiveness of Social Problem-Solving Interventions

This three-level meta-analysis synthesized 21 SCED studies examining SPS and SPS-component intervention packages for individuals with autism. The unconditional three-level meta-analytic model estimated a positive and statistically significant pooled effect of 1.741, indicating that SPS-related outcomes improved from baseline to intervention phases. Because many included studies evaluated multicomponent intervention packages, the observed effects cannot be attributed specifically to SPS components. Instead, the pooled estimate reflects the combined impact of intervention packages in which SPS strategies were embedded alongside other instructional, behavioral, or contextual components, making it impossible to isolate or quantify the independent contribution of SPS within the available SCED evidence. Accordingly, any interpretation of SPS as the sole active mechanism should be avoided, as the current evidence base does not allow disentangling SPS-specific effects from co-occurring intervention components such as prompting, modeling, reinforcement, or self-management strategies.

The pooled estimate should be interpreted within the SCED context. It reflects standardized baseline-to-intervention phase change and may be influenced by baseline variability, phase length, baseline trend, and serial dependence. Therefore, it should not be interpreted in the same way as a conventional group-design standardized mean difference (Cheung, 2013; Van den Noortgate et al., 2013). Rather, the result suggests that SPS-related intervention packages were associated with positive baseline-to-intervention changes in targeted outcomes across the included SCED studies.

These findings are consistent with prior syntheses showing positive effects of social-skills-related interventions for individuals with autism (Wang et al., 2013), while extending the evidence base to interventions that explicitly taught or embedded SPS processes (Bellini et al., 2007; Gates et al., 2017). The findings also complement recent group-design evidence showing positive effects of SPS interventions on SPS competence and related social–emotional outcomes (Jin et al., 2025). Together, these bodies of evidence suggest that SPS is a promising intervention focus, although SCED and group-design findings should not be directly compared because they differ in design, measurement, and analytic assumptions.

Overall, the present results support immediate baseline-to-intervention improvements in targeted SPS-related outcomes following SPS and SPS-component intervention packages. From a practical and educational perspective, these improvements may reflect functional gains in how individuals with autism respond to socially relevant problem situations, including identifying problems, generating alternative responses, and applying structured decision-making strategies within supported contexts. In applied settings, such changes may translate into increased independence in peer interactions, reduced reliance on adult prompting, and improved adaptive responding in classroom, home, and community environments. However, these interpretations should be considered cautiously, as the current evidence is based on SCED baseline-to-intervention comparisons and does not provide direct evidence regarding long-term maintenance or generalization. Future research should further examine the durability and contextual generalization of SPS-related intervention effects.

### 4.2. Moderating Effects of Study Characteristics

The moderator analyses did not identify clear statistically significant predictors of effect-size variability. SPS centrality did not significantly moderate effect sizes; however, this finding should be interpreted as a lack of statistically detectable differences rather than evidence of equivalence across Comprehensive SPS, Functional problem-solving, and Embedded SPS-component interventions (McNair et al., 2024). This finding is important because it indicates that positive baseline-to-intervention changes were observed not only in studies that taught a relatively complete SPS process, but also in studies that taught problem solving in constrained functional contexts or embedded selected SPS components within broader intervention packages (Cote et al., 2014; McLucas et al., 2026; Stauch & Plavnick, 2020; Villante et al., 2021). However, this result should not be interpreted as evidence that these intervention categories are equivalent. The number of studies within each category was small, and the SPS centrality classification was intended to clarify construct heterogeneity rather than to establish comparative intervention efficacy.

Intervention setting also did not reach statistical significance, although the model-based estimates were numerically higher for school-based and home-based interventions than for clinical/community/transition settings. This pattern is consistent with the possibility that naturalistic contexts may provide more frequent opportunities for practicing SPS-related skills during everyday routines, peer interactions, and functional problem situations (Rotheram-Fuller et al., 2010; Kasari & Patterson, 2012; Whalon et al., 2015). At the same time, because the omnibus moderator test was not statistically significant, these differences should be interpreted as descriptive trends rather than evidence that setting systematically moderates SPS intervention effects (Cheung, 2013; Van den Noortgate et al., 2013). The small number of home-based studies further limits confidence in the stability of this subgroup estimate.

WWC methodological quality rating was at the conventional significance threshold and was interpreted cautiously. Studies that met WWC standards without reservations showed numerically higher estimates than studies that met standards with reservations (What Works Clearinghouse, 2020; Wang et al., 2013). This pattern may reflect the possibility that stronger SCED features, clearer phase documentation, and more consistent demonstrations of experimental control are associated with more stable intervention effects. However, the finding should not be treated as confirmatory, especially given the exploratory nature of the moderator analyses and the limited number of independent studies.

No clear moderation effects were found for implementer type, intervention type, participant age, or participant gender composition. These findings suggest that positive outcomes were observed across different delivery agents, intervention formats, and participant characteristics (Wang et al., 2013; Whalon et al., 2015). However, small subgroup sizes and inconsistent reporting of participant characteristics limited the power and precision of these analyses.

Given the relatively small number of studies and limited sample sizes within moderator subgroups, the statistical power to detect moderation effects was likely constrained. Therefore, all moderator findings should be considered exploratory and interpreted with caution. Future SCED studies should report intervention components, implementer roles, instructional contexts, participant characteristics, and maintenance or generalization outcomes more consistently so that future syntheses can better examine which conditions are associated with stronger SPS-related outcomes.

### 4.3. Implications for Research and Practice

#### 4.3.1. Theoretical Implications

The present findings contribute to the growing conceptual distinction between SPS interventions and broader social skills training approaches in autism intervention research. Traditional social skills interventions often emphasize the acquisition or rehearsal of observable behavioral repertoires, whereas SPS interventions place greater emphasis on the cognitive and metacognitive processes involved in adaptive social functioning (D’Zurilla et al., 2004; Nezu, 2004). These processes include identifying social or functional problems, interpreting contextual cues, generating alternative responses, evaluating consequences, and selecting or implementing adaptive actions (Crick & Dodge, 1994; Pellicano, 2012). This distinction is consistent with theoretical perspectives that conceptualize social behavior as an active problem-solving process involving social information processing, emotional regulation, executive functioning, flexible thinking, and behavioral decision making (Szumski et al., 2019; Bamicha & Drigas, 2022; Bonete et al., 2022; McNair et al., 2024).

At the same time, the present findings should not be interpreted as evidence that SPS operates through a single mechanism or that SPS components alone caused the observed effects. Many included studies evaluated multicomponent intervention packages that combined SPS-related instruction with modeling, reinforcement, prompting, role-play, visual supports, social narratives, self-monitoring procedures, or technology-based supports (Bock, 2007; Dotto-Fojut et al., 2011; Suarez et al., 2022). These combinations are common and useful in applied intervention contexts, but they make it difficult to determine which components were most responsible for behavioral change. Future research should therefore specify SPS components more clearly and examine whether comprehensive SPS instruction differs from interventions that embed selected SPS components within broader instructional packages.

The non-significant effect of SPS centrality is also theoretically informative, but it should be interpreted cautiously. Positive baseline-to-intervention changes were observed across Comprehensive SPS, Functional problem-solving, and Embedded SPS-component interventions, suggesting that SPS-related processes may be useful across a range of intervention formats. However, the absence of a significant moderator effect should not be taken as evidence that these categories are equivalent (McNair et al., 2024). Rather, the classification highlights the need for clearer construct boundaries in future research. Studies should explicitly state whether SPS is the primary instructional target or an embedded component, and should identify the specific problem-solving steps taught to participants.

Finally, the present review demonstrates the methodological value of three-level meta-analytic models for synthesizing SCED evidence in autism intervention research. SCED studies often generate multiple dependent-variable-level outcomes nested within participants and studies (Hedges et al., 2012; Wang et al., 2013). Three-level modeling provides a way to retain this information while accounting for dependency among effect sizes. In the present review, this approach allowed the synthesis to estimate the overall baseline-to-intervention change while also partitioning heterogeneity across study and participant cluster levels.

#### 4.3.2. Practical Implications

The findings from this meta-analysis suggest that SPS intervention may be useful for improving targeted SPS-related outcomes for individuals with autism. Practitioners should interpret this conclusion as evidence for structured intervention packages that include SPS-related instruction, rather than as evidence for any single isolated SPS component (McNair et al., 2024). In practice, SPS instruction may be particularly useful when it is explicit, structured, and supported by concrete teaching procedures such as modeling, visual cues, guided rehearsal, prompting, feedback, reinforcement, and self-monitoring.

Although intervention setting was not a statistically significant moderator, school-based and home-based interventions showed numerically higher estimates than clinical/community/transition settings. This pattern suggests that naturalistic contexts may provide meaningful opportunities for practicing SPS-related skills during everyday routines, peer interactions, and functional problem situations. School contexts may be especially relevant because they provide repeated opportunities for authentic peer interaction, contextualized social problem solving, and immediate feedback within naturally occurring activities (Rotheram-Fuller et al., 2010; Kasari & Patterson, 2012; Whalon et al., 2015). However, these differences should be interpreted as descriptive trends rather than evidence that one setting is clearly superior.

Teachers and school staff may benefit from training that goes beyond general behavior management and includes explicit instruction in social reasoning and problem-solving processes (Bellini et al., 2007; Gates et al., 2017). SPS steps can be adapted into visual supports, self-monitoring checklists, role-play activities, social narratives, or digital applications. For example, structured sequences such as Stop-Observe-Deliberate-Act can help learners identify a problem, consider possible responses, and select an adaptive action (Bock, 2007). Such procedures may be especially helpful when they are individualized to learners’ communication abilities, cognitive profiles, and support needs.

The absence of clear moderation by implementer type suggests that SPS-related intervention packages may be adaptable across different delivery agents, including educators, therapists, paraprofessionals, researchers, and caregivers. However, this finding should not be interpreted to mean that implementer training is unnecessary or that all implementers are equally effective under all conditions. Effective implementation likely depends on clear instructional procedures, adequate coaching, fidelity monitoring, and opportunities for repeated practice across meaningful contexts.

SPS interventions may also have practical relevance for transition-age adolescents and young adults. Several included studies targeted vocational, community, or functional problem-solving outcomes, highlighting the potential value of SPS instruction beyond traditional social-skills training (Stauch & Plavnick, 2020; McLucas & Gonçalves, 2025). Overall, practitioners should tailor SPS-related intervention packages to learners’ communication profiles, developmental levels, support needs, and ecological demands while maintaining explicit instruction in the core problem-solving process.

### 4.4. Limitations and Future Directions

Several limitations should be considered when interpreting the present findings. First, although the final dataset included 114 DV-level effect sizes nested within 59 participant clusters and 21 studies, the independent SCED literature on SPS and SPS-component interventions remains relatively limited. Several moderator categories included small numbers of studies or effect sizes, which reduced statistical power and limited the stability of subgroup estimates.

Second, the included interventions varied in the extent to which SPS was the central instructional target. Some studies taught a comprehensive SPS sequence, whereas others targeted functional problem solving or embedded selected SPS components within broader intervention packages. Although SPS centrality was coded to address this construct heterogeneity, the multi-component nature of many interventions makes it difficult to isolate the unique contribution of SPS components. Future research should define SPS components more explicitly and compare comprehensive SPS instruction with embedded SPS-component approaches.

Third, the present analysis focused on baseline-to-intervention changes in targeted outcomes. Maintenance, follow-up, and generalization data were not included in the primary effect-size estimates in order to improve comparability across studies. As a result, the durability and transfer of SPS-related gains remain uncertain. Future SCED studies should more systematically evaluate whether gains are maintained over time and generalized across settings, communication partners, materials, and naturally occurring social or functional problems.

Fourth, publication-bias analyses should be interpreted cautiously. Funnel plot inspection, Egger’s regression test, Rosenthal’s fail-safe N, and trim-and-fill procedures were conducted at the study level because conventional diagnostics assume independent effect sizes. However, publication bias and selective outcome reporting are recognized concerns in applied behavior analysis, SCED, and special education meta-analyses, and the limited number of independent studies and dependent structure of the present SCED data mean that these risks cannot be fully ruled out (Dowdy et al., 2021; Gage et al., 2017; Tincani & Travers, 2019).

Finally, participant characteristics and contextual variables were not consistently reported across primary studies. Important factors such as language ability, cognitive functioning, adaptive behavior, co-occurring conditions, cultural context, and support needs could not be examined systematically (Wang et al., 2013; Whalon et al., 2015). Future research should prioritize clearer reporting, larger multi-site SCED replications, preregistered protocols, component analyses, and stronger documentation of maintenance and generalization outcomes. Technology-mediated SPS interventions, including virtual reality, digital self-monitoring, and individualized coaching systems, may also offer promising directions, but their effectiveness should be evaluated through rigorous SCEDs with transparent reporting and fidelity assessment.

## 5. Conclusions

This three-level meta-analysis synthesized evidence from 21 SCED studies examining SPS interventions for individuals with autism. The findings showed a positive and statistically significant pooled effect, indicating that interventions incorporating SPS-related instruction were associated with improvements in targeted social, communicative, functional, and adaptive outcomes from baseline to intervention phases. Because many included studies evaluated multicomponent intervention packages, the pooled estimate reflects the combined effects of interventions in which SPS-related components were embedded, rather than the independent effect of SPS components in isolation. Moderator analyses did not identify clear and statistically detectable predictors of effect-size variability across SPS centrality, setting, intervention type, implementer type, participant age, participant gender composition, or WWC methodological quality rating. Therefore, no firm conclusions can be drawn regarding differential effectiveness across intervention or participant characteristics given the limited statistical power and exploratory nature of these analyses. Future research should define SPS components more explicitly, report participant and intervention characteristics more consistently, and examine whether SPS-related gains are maintained over time and generalized across real-life contexts.

## Figures and Tables

**Table 1:** Three-Level Effect Size Distribution and Variance Components.

Level	*k*	Mean *ES*	*SD*	Range	Variance Component	*I*^2^ (%)
Level 3: Study	21	1.78	0.35	0.97–2.45	0.0749	-
Level 2: Participant	59	1.79	0.38	0.79–2.49	0.0227	-
Level 1: DV	114	1.84	0.49	0.50–3.89	-	-
Total heterogeneity	-	-	-	-	-	24.48

**Notes.** *k* = number of effect-size estimates at the corresponding level. *SD* = standard deviation. *ES* = effect-size estimate. DV = dependent variable. Participant clusters were nested within studies. The total *I*^2^ value summarizes the approximate proportion of total variability attributable to heterogeneity in the three-level model.

**Table 2:** Moderator Analyses From Three-Level Meta-Analytic Models.

Moderator	*k*	Estimate	*SE*	QM/z	df	*p*	95% *CI*	*τ* ^2^_3_	Δ*τ* ^2^_3_
SPS centrality				QM(2) = 0.58	2	0.747		0.086	+15.2%
Comprehensive SPS interventions	34	1.85	0.18	10.28	-	<0.001	[1.50, 2.21]		
Functional problem-solving interventions	30	1.68	0.15	10.66	-	<0.001	[1.37, 1.99]		
Embedded SPS-component interventions	50	1.71	0.13	12.33	-	<0.001	[1.44, 1.98]		
Intervention setting				QM(2) = 5.55	2	0.062		0.048	−35.6%
Clinical/community/transition	29	1.44	0.16	9.01	-	<0.001	[1.13, 1.76]		
School-based	76	1.80	0.09	18.94	-	<0.001	[1.61, 1.98]		
Home-based	9	2.11	0.27	7.73	-	<0.001	[1.57, 2.64]		
WWC quality rating				QM(1) = 3.85	1	0.050		0.048	−35.5%
Score = 2	34	1.49	0.14	10.05	-	<0.001	[1.20, 1.78]		
Score = 3	80	1.84	0.09	19.85	-	<0.001	[1.65, 2.02]		
Implementer type				QM(2) = 0.91	2	0.635		0.082	+9.2%
Therapist/behavior technician	32	1.82	0.18	10.00	-	<0.001	[1.46, 2.18]		
Researcher	69	1.75	0.11	15.57	-	<0.001	[1.53, 1.97]		
Educator/paraprofessional	13	1.53	0.24	6.26	-	<0.001	[1.05, 2.01]		
Intervention type				QM(5) = 2.99	5	0.701		0.081	+7.7%
Combined	32	1.55	0.16	9.44	-	<0.001	[1.23, 1.88]		
Direct problem-solving training	47	1.89	0.13	14.46	-	<0.001	[1.64, 2.15]		
Technology-based	11	1.63	0.29	5.60	-	<0.001	[1.06, 2.20]		
Video modeling	18	1.66	0.26	6.21	-	<0.001	[1.14, 2.19]		
Social story	3	1.80	0.51	3.52	-	<0.001	[0.79, 2.80]		
Other	3	1.58	0.52	3.03	-	0.002	[0.56, 2.60]		
Participant age	114	−0.01	0.01	QM(1) = 0.27	1	0.606	[−0.04, 0.02]	0.080	+6.2%
Participant gender composition	114	0.07	0.26	QM(1) = 0.08	1	0.773	[−0.44, 0.59]	0.073	−2.1%

**Note.** Estimate = model-based subgroup estimate for categorical moderators and regression coefficient for continuous moderators; *k* = number of DV-level effect sizes; *CI* = confidence interval. Omnibus effects were tested using Wald-type QM tests, and category-specific estimates were tested using Wald z tests. *τ*^2^_3_ represents residual between-study variance after inclusion of the moderator, and Δ*τ*^2^_3_ represents its proportional change relative to the unconditional model (*τ*^2^_3_ = 0.0749). SPS = social problem solving; WWC = What Works Clearinghouse.

## Data Availability

The data supporting the findings of this study are available from the corresponding author upon reasonable request. Supplementary datasets, coding protocols, and analysis scripts are provided in the Supplementary Materials.

## Supplementary Materials

The following supporting information can be downloaded at https://www.mdpi.com/article/10.3390/bs16071129/s1, Table S1: Detailed search strategies for each database. Materials S1: Full references of all studies included in qualitative analysis. Table S2: PRISMA 2020 Checklist for the Meta-analysis; Table S3: Risk of Bias Assessments Using What Works Clearinghouse Single-Case Design Standards; Table S4: Study, Participant, Study-Level, and DV/Effect-Size Characteristics of Included Studies.
