# Supplementary material for: Single-Case Experimental Evidence on Social Problem-Solving Interventions for Individuals with Autism: A Three-Level Meta-Analysis
Source: Behav Sci (Basel). 2026 Jul 6;16(7):1129. doi: 10.3390/bs16071129 (PMC13405220; doi:10.3390/bs16071129)
Supplement: Supplementary file 1 [file behavsci-16-01129-s001.zip › behavsci-4359797-supplementary.pdf]

**Supplemental Table S1. Detailed search strategies for each database.**

| Database | Search Strategy | Results |
|---|---|---|
| Web of Science | TS=("autism" OR "autism spectrum disorder" OR "ASD" OR "autistic" OR "Asperger" OR "pervasive developmental disorder") AND TS=("problem solving" OR "problem-solving" OR "social problem" OR "conflict resolution" OR "social decision making" ) AND TS=("intervention" OR "training" OR "teaching" OR "instruction" OR "treatment" OR "program" OR "therapy") AND TS=("single case" OR "single-case" OR "single subject" OR "single-subject" OR "multiple baseline") | 78 |
| PubMed | ((("autism spectrum disorder"[MeSH Terms] OR "autism"[tiab] OR "autism spectrum disorder"[tiab] OR "ASD"[tiab] OR "autistic"[tiab] OR "Asperger"[tiab] OR "pervasive developmental disorder"[tiab])) AND (("problem solving"[MeSH Terms] OR "problem-solving"[tiab] OR "problem solving"[tiab] OR "social problem"[tiab] OR "conflict resolution"[tiab] OR "social decision"[tiab])) AND (("single case"[tiab] OR "single-case"[tiab] OR "single subject"[tiab] OR "single-subject"[tiab] OR "multiple baseline"[tiab] ))) | 46 |
| PsycINFO | (DE "Autistic Disorder" OR DE "Autism Spectrum Disorders" OR "autism" OR "ASD" OR "autistic" OR "Asperger" OR "pervasive developmental disorder") AND (DE "Problem Solving" OR "problem solv*" OR "social problem*" OR "conflict resolution" OR "social decision*") AND (DE "Intervention" OR DE "Training" OR"intervention" OR "training" OR "teaching" OR "instruction" OR "treatment" OR "program" OR "therapy") AND (DE "Single Case Design" OR "single case" OR "single-case" OR "single subject" OR "single-subject" OR "multiple baseline") | 112 |
| Scopus | TITLE-ABS-KEY("autism spectrum disorder" OR "autism" OR "ASD" OR "autistic" OR "Asperger" OR "pervasive developmental disorder") AND TITLE-ABS-KEY("problem solving" OR "social problem solving" OR "social problem" OR "conflict resolution" OR "social decision") AND TITLE-ABS-KEY("intervention" OR "training" OR "teaching" OR "instruction" OR "treatment" OR "program" OR "therapy") AND TITLE-ABS-KEY("single case design" OR "single case" OR "single-case" OR "single subject" OR "single-subject" OR "multiple baseline") | 48 |
| SpringerLink | ("autism" OR "autism spectrum disorder" OR autistic OR ASD) AND ("social problem solving" OR "social problem-solving" OR "interpersonal problem solving" OR "social conflict resolution") AND (intervention OR training OR teaching OR instruction) AND ("single-case" OR "single subject" OR "single-subject" OR "multiple baseline") | 54 |
| ERIC | (descriptor:"Autism Spectrum Disorders" OR descriptor:"Autism" OR title,abstract:"autism" OR title,abstract:"ASD" OR title,abstract:"autistic" OR title,abstract:"Asperger") AND (descriptor:"social problem solving" OR title,abstract:"problem-solving*" OR title,abstract:"social problem*" OR title,abstract:"conflict resolution" ) AND (descriptor:"Intervention" OR descriptor:"Training" OR title,abstract:"intervention" OR title,abstract:"training" OR title,abstract:"teaching" OR title,abstract:"instruction" OR title,abstract:"treatment" OR title,abstract:"program" OR title,abstract:"therapy") AND | 98 |

| | | |
|---|---|---|
| | (descriptor:"Single Subject Research" OR title,abstract:"single case" OR title,abstract:"single-case" OR title,abstract:"single subject" OR title,abstract:"multiple baseline") | |
| Total | | 436 |

**Supplementary Materials S1. Full references of all studies included in qualitative analysis.**


Agran, M., Blanchard, C., Wehmeyer, M., & Hughes, C. (2002). Increasing the problem-solving skills of students with developmental disabilities participating in general education. *Remedial and Special Education*, *23*(5), 279–288. https://doi.org/10.1177/07419325020230050301

Alzrayer, N. M., Banda, D. R., & Koul, R. K. (2019). The effects of systematic instruction in teaching multistep social-communication skills to children with autism spectrum disorder using an iPad. *Developmental Neurorehabilitation*, *22*(6), 415–429. https://doi.org/10.1080/17518423.2019.1604578

Bock, M. A. (2007a). A social behavioral learning strategy intervention for a child with asperger syndrome: Brief report. *Remedial and Special Education*, *28*(5), 258–265. https://doi.org/10.1177/07419325070280050101

Bock, M. A. (2007b). The impact of social Behavioral learning strategy training on the social interaction skills of four students with asperger syndrome. *Focus on Autism and Other Developmental Disabilities*, *22*(2), 88–95. https://doi.org/10.1177/10883576070220020901

Cote, D. L., Jones, V. L., Barnett, C., Pavelek, K., Nguyen, H., & Sparks, S. L. (2014). Teaching problem solving skills to elementary age students with autism. *Education and Training in Autism and Developmental Disabilities*, *49*(2), 189–199.

Cox, S. K., Root, J. R., McConomy, A., & Davis, K. (2024). “for whom” and “under what conditions” is MSBI effective? A conceptual replication with high school students with autism. *Exceptional Children*, *90*(4), 361–381. https://doi.org/10.1177/00144029241259013

Davis, K. M., Boon, R. T., Cihak, D. F., & ForeIII, C. (2010). Power cards to improve conversational skills in adolescents with asperger syndrome. *Focus on Autism and Other Developmental Disabilities*, *25*(1), 12–22. https://doi.org/10.1177/1088357609354299

Dotto-Fojut, K. M., Reeve, K. F., Townsend, D. B., & Progar, P. R. (2011). Teaching adolescents with autism to describe a problem and request assistance during simulated vocational tasks. *Research in Autism Spectrum Disorders*, *5*(2), 826–833. https://doi.org/10.1016/j.rasd.2010.09.012

Frampton, S. E., & Axe, J. B. (2025). A preliminary investigation into teaching adolescents with autism to use apps to solve problems. *The Analysis of Verbal Behavior*, *41*(1), 26–39. https://doi.org/10.1007/s40616-024-00212-8

Ingvarsson, E. T., & Hollobaugh, T. (2010). Acquisition of intraverbal behavior: Teaching children with autism to mand for answers to questions. *Journal of Applied Behavior Analysis*, *43*(1), 1–

17. https://doi.org/10.1901/jaba.2010.43-1

Keesey-Phelan, S. H., Axe, J. B., & Williams, A. L. (2022). The effects of teaching a problem-solving strategy on recalling past events with a child with autism. *The Analysis of Verbal Behavior*, *38*(2), 191–198. https://doi.org/10.1007/s40616-022-00176-7

Lora, C. C., Kisamore, A. N., Reeve, K. F., & Townsend, D. B. (2020). Effects of a problem-solving strategy on the independent completion of vocational tasks by adolescents with autism spectrum disorder. *Journal of Applied Behavior Analysis*, *53*(1), 175–187. https://doi.org/10.1002/jaba.558

Marckel, J. M., Neef, N. A., & Ferreri, S. J. (2006). A preliminary analysis of teaching improvisation with the picture exchange communication system to children with autism. *Journal of Applied Behavior Analysis*, *39*(1), 109–115. https://doi.org/10.1901/jaba.2006.131-04

McLucas, A. S., & Gonçalves, B. F. (2025). Using practitioner-implemented video models to teach vocational social skills in a rural public school. *Career Development and Transition for Exceptional Individuals*, 21651434251328271. https://doi.org/10.1177/21651434251328271

McLucas, A. S., Som, S., Fleming, J., Ingvarsson, E., & Therrien, W. J. (2024). Using video modeling plus feedback to teach vocational social skills to employment-aged autistic youth. *Journal of Behavioral Education*, 1–24. https://doi.org/10.1007/s10864-024-09561-9

Root, J. R., Cox, S. K., Davis, K., & Gonzales, S. (2022). Using augmented reality and modified schema-based instruction to teach problem solving to students with autism. *Remedial and Special Education*, *43*(5), 301–313. https://doi.org/10.1177/07419325211054209

Stauch, T. A., & Plavnick, J. B. (2020). Teaching vocational and social skills to adolescents with autism using video modeling. *Education and Treatment of Children*, *43*(2), 137–151. https://doi.org/10.1007/s43494-020-00020-4

Suarez, V. D., Najdowski, A. C., Tarbox, J., Moon, E., St. Clair, M., & Farag, P. (2022). Teaching individuals with autism problem-solving skills for resolving social conflicts. *Behavior Analysis in Practice*, *15*(3), 768–781. https://doi.org/10.1007/s40617-021-00643-y

Villante, N. K., Lerman, D. C., Som, S., & Hunt, J. C. (2021). Teaching adults with developmental disabilities to problem solve using electronic flowcharts in a simulated vocational setting. *Journal of Applied Behavior Analysis*, *54*(3), 1199–1219. https://doi.org/10.1002/jaba.786

Yakubova, G., & Taber-Doughty, T. (2017). Improving problem-solving performance of students with autism spectrum disorders. *Focus on Autism and Other Developmental Disabilities*, *32*(1), 3–17. https://doi.org/10.1177/1088357615587506

Yakubova, G., & Zeleke, W. A. (2016). A problem-solving intervention using iPads to improve transition-related task performance of students with autism spectrum disorder. *Journal of Special Education Technology*, *31*(2), 77–86. https://doi.org/10.1177/0162643416650023

**Supplemental Table S2. PRISMA 2020 Checklist for the Present Systematic Review and Three-Level Meta-Analysis.**

| Section and Topic | Item # | Checklist item | Location where item is reported |
|---|---|---|---|
| **TITLE** | | | |
| Title | 1 | Identify the report as a systematic review. | Title: "Single-Case Experimental Evidence on Social Problem-Solving Interventions for Individuals with Autism: A Three-Level Meta-Analysis" |
| **ABSTRACT** | | | |
| Abstract | 2 | See the PRISMA 2020 for Abstracts checklist. | Abstract |
| **INTRODUCTION** | | | |
| Rationale | 3 | Describe the rationale for the review in the context of existing knowledge. | Introduction, paragraphs describing SPS intervention research gap and need for SCED synthesis. |
| Objectives | 4 | Provide an explicit statement of the objective(s) or question(s) the review addresses. | Introduction, final paragraph / study purpose statement. |
| **METHODS** | | | |
| Eligibility criteria | 5 | Specify the inclusion and exclusion criteria for the review and how studies were grouped for the syntheses. | Methods: Inclusion and Exclusion Criteria; Coding of study characteristics. |
| Information sources | 6 | Specify all databases, registers, websites, organizations, reference lists and other sources searched or consulted to identify studies. Specify the date when each source was last searched or consulted. | Methods: Search Strategy / Literature Search |
| Search strategy | 7 | Present the full search strategies for all databases, registers and websites, including any filters and limits used. | Methods: Search Strategy; Supplementary Materials: Table S1 / Appendix (full search strings). |
| Selection process | 8 | Specify the methods used to decide whether a study met the inclusion criteria of the review, including how many reviewers screened each record and each report retrieved, whether they worked independently, and if applicable, details of automation tools used in the process. | Methods: Study Selection |
| Data collection | 9 | Specify the methods used to collect data from reports, including how many reviewers collected data from | Methods: Data Extraction and Coding;Supplementary Materials: |

| Section and Topic | Item # | Checklist item | Location where item is reported |
|---|---|---|---|
| process | | each report, whether they worked independently, any processes for obtaining or confirming data from study investigators, and if applicable, details of automation tools used in the process. | Table S3 |
| Data items | 10a | List and define all outcomes for which data were sought. Specify whether all results that were compatible with each outcome domain in each study were sought (e.g. for all measures, time points, analyses), and if not, the methods used to decide which results to collect. | Methods: Data Extraction and Coding; outcome definition section |
| | 10b | List and define all other variables for which data were sought (e.g. participant and intervention characteristics, funding sources). Describe any assumptions made about any missing or unclear information. | Methods: Data Extraction and Coding; moderator coding section |
| Study risk of bias assessment | 11 | Specify the methods used to assess risk of bias in the included studies, including details of the tool(s) used, how many reviewers assessed each study and whether they worked independently, and if applicable, details of automation tools used in the process. | Methods: Risk of Bias Assessment |
| Effect measures | 12 | Specify for each outcome the effect measure(s) (e.g. risk ratio, mean difference) used in the synthesis or presentation of results. | Methods: Statistical Analysis (SMD) |
| Synthesis methods | 13a | Describe the processes used to decide which studies were eligible for each synthesis (e.g. tabulating the study intervention characteristics and comparing against the planned groups for each synthesis (item #5)). | Methods: Statistical Analysis / Effect Size Calculation |
| | 13b | Describe any methods required to prepare the data for presentation or synthesis, such as handling of missing summary statistics, or data conversions. | Methods: Effect Size Calculation; Data Preparation |
| | 13c | Describe any methods used to tabulate or visually display results of individual studies and syntheses. | Methods: Statistical Analysis |
| | 13d | Describe any methods used to synthesize results and provide a rationale for the choice(s). If meta-analysis was performed, describe the model(s), method(s) to identify the presence and extent of statistical heterogeneity, and software package(s) used. | Methods: Three-Level Meta-Analytic Model; Heterogeneity Analysis; Statistical software section |
| | 13e | Describe any methods used to explore possible causes of heterogeneity among study results (e.g. subgroup analysis, meta-regression). | Methods: Moderator Analyses |

| Section and Topic | Item # | Checklist item | Location where item is reported |
|---|---|---|---|
| | 13f | Describe any sensitivity analyses conducted to assess robustness of the synthesized results. | Methods: Sensitivity Analysis |
| Reporting bias assessment | 14 | Describe any methods used to assess risk of bias due to missing results in a synthesis (arising from reporting biases). | Methods: Reporting Bias Assessment (Funnel plot, Egger's test, Trim-and-fill) |
| Certainty assessment | 15 | Describe any methods used to assess certainty (or confidence) in the body of evidence for an outcome. | [Not explicitly stated using formal method] |
| **RESULTS** | | | |
| Study selection | 16a | Describe the results of the search and selection process, from the number of records identified in the search to the number of studies included in the review, ideally using a flow diagram. | Results: Study Selection; Figure 1 (PRISMA Flow Diagram) |
| | 16b | Cite studies that might appear to meet the inclusion criteria, but which were excluded, and explain why they were excluded. | Results: Study Selection / full-text exclusion reasons |
| Study characteristics | 17 | Cite each included study and present its characteristics. | Results: Characteristics of Included Studies section |
| Risk of bias in studies | 18 | Present assessments of risk of bias for each included study. | Results: Risk of Bias within Studies; Supplementary Table S2 |
| Results of individual studies | 19 | For all outcomes, present, for each study: (a) summary statistics for each group (where appropriate) and (b) an effect estimates and its precision (e.g. confidence/credible interval), ideally using structured tables or plots. | Results: Overall Effect Size; Figure 2 |
| Results of syntheses | 20a | For each synthesis, briefly summaries the characteristics and risk of bias among contributing studies. | Results: Overall Effect Size / Moderator Analyses |
| | 20b | Present results of all statistical syntheses conducted. If meta-analysis was done, present for each the summary estimate and its precision (e.g. confidence/credible interval) and measures of statistical heterogeneity. If comparing groups, describe the direction of the effect. | Results: Overall Effect Size; Variance Components and Heterogeneity |
| | 20c | Present results of all investigations of possible causes of heterogeneity among study results. | Results: Moderator Analyses; Table 2 |
| | 20d | Present results of all sensitivity analyses conducted to assess the robustness of the synthesized results. | Results: Sensitivity Analysis and Publication Bias |
| Reporting biases | 21 | Present assessments of risk of bias due to missing results (arising from reporting biases) for each synthesis assessed. | Results: Sensitivity Analysis and Publication Bias; Figure 3 |

| Section and Topic | Item # | Checklist item | Location where item is reported |
|---|---|---|---|
| Certainty of evidence | 22 | Present assessments of certainty (or confidence) in the body of evidence for each outcome assessed. | Not assessed |
| **DISCUSSION** | | | |
| Discussion | 23a | Provide a general interpretation of the results in the context of other evidence. | Discussion: "Overall Effectiveness of Social Problem-Solving Interventions," |
| | 23b | Discuss any limitations of the evidence included in the review. | Discussion: Limitations and Future Directions |
| | 23c | Discuss any limitations of the review processes used. | Discussion: Limitations and Future Directions |
| | 23d | Discuss implications of the results for practice, policy, and future research. | Discussion: Implications for Research and Practice |
| **OTHER INFORMATION** | | | |
| Registration and protocol | 24a | Provide registration information for the review, including register name and registration number, or state that the review was not registered. | Methods: "Search Strategy"; OSF registration: https://osf.io/zex2q |
| | 24b | Indicate where the review protocol can be accessed, or state that a protocol was not prepared. | Methods: Protocol and Registration; OSF record / Supplementary Materials |
| | 24c | Describe and explain any amendments to information provided at registration or in the protocol. | Methods: Protocol and Registration |
| Support | 25 | Describe sources of financial or non-financial support for the review, and the role of the funders or sponsors in the review. | Acknowledgments / Funding statement |
| Competing interests | 26 | Declare any competing interests of review authors. | Competing Interests statement |
| Availability of data, code and other materials | 27 | Report which of the following are publicly available and where they can be found: template data collection forms; data extracted from included studies; data used for all analyses; analytic code; any other materials used in the review. | Data Availability Statement; Supplementary Materials |

*From:* Page MJ, McKenzie JE, Bossuyt PM, Boutron I, Hoffmann TC, Mulrow CD, et al. The PRISMA 2020 statement: an updated guideline for reporting systematic reviews. BMJ 2021;372:n71. doi: 10.1136/bmj.n71. This work is licensed under CC BY 4.0. To view a copy of this license, visit https://creativecommons.org/licenses/by/4.0/

**Supplemental Table S3. Risk of Bias Assessments Using What Works Clearinghouse Single-Case Design Standards.**

| Author (Year) | QI1 | QI2 | QI3 | QI4 | QI5 | Overall Rating | WWC Score |
|---|---|---|---|---|---|---|---|
| Bock (2007)a | Y | Y | Y | Y | Y | Meets Standards | 3 |
| Bock (2007)b | Y | Y | Y | Y | Y | Meets Standards | 3 |
| Dotto-Fojut et al. (2011) | Y | Y | Y | Y | Y | Meets Standards | 3 |
| Cote et al. (2014) | Y | Y | Y | Y | Y | Meets Standards | 3 |
| Lora et al. (2020) | Y | Y | Y | Y | Y | Meets Standards | 3 |
| McLucas et al. (2024) | Y | Y | Y | Y | Y | Meets With Reservations | 2 |
| McLucas & Goncalves (2025) | Y | Y | Y | Y | Y | Meets Standards | 3 |
| Stauch & Plavnick (2020) | Y | Y | Y | Y | Y | Meets With Reservations | 2 |
| Root et al. (2022) | Y | Y | Y | Y | Y | Meets Standards | 3 |
| Yakubova & Taber-Doughty (2017) | Y | Y | Y | Y | Y | Meets Standards | 3 |
| Keesey-Phelan et al. (2022) | Y | Y | Y | Y | Y | Meets With Reservations | 2 |
| Suarez et al. (2022) | Y | Y | Y | Y | Y | Meets With Reservations | 2 |
| Yakubova & Zeleke (2016) | Y | Y | Y | Y | Y | Meets With Reservations | 2 |
| Villante et al. (2021) | Y | Y | Y | Y | Y | Meets Standards | 3 |
| Agran et al. (2002) | Y | Y | Y | Y | Y | Meets Standards | 3 |
| Frampton & Axe (2025) | Y | Y | Y | Y | Y | Meets With Reservations | 2 |
| Davis et al. (2010) | Y | Y | Y | Y | Y | Meets Standards | 3 |
| Marckel et al. (2006) | Y | Y | Y | Y | Y | Meets Standards | 3 |
| Alzrayer et al. (2019) | Y | Y | Y | Y | Y | Meets Standards | 3 |
| Cox et al. (2024) | Y | Y | Y | Y | Y | Meets Standards | 3 |
| Ingvarsson & Hollobaugh (2010) | Y | Y | Y | Y | Y | Meets Standards | 3 |

***Note.*** Y = Yes (criterion met); N = No (criterion not met); N/A = Not Applicable. Checklist Items: QI = quality indicator per Wang and Parrila (2008) and What Works Clearinghouse (2022) standards: QI1 = sufficient

participant information (diagnosis confirmation, age, ability measures); QI2 = detailed settings/materials description; QI3 = replicable intervention procedures; QI4 = operationally defined dependent variables with clear measurement protocols; QI5 = use of multiple-baseline or reversal design. Overall Rating: Meets Standards = no reservations (score = 3); Meets With Reservations = minor concerns (score = 2); Does Not Meet Standards = major concerns (score = 1; none in final sample).

**Supplemental Table S4. Study, Participant, Study-Level, and DV/Effect-Size Characteristics of Included Studies.**

| Study | Participant characteristics | | | Study characteristics | | | | | DV/effect-size characteristics | |
|---|---|---|---|---|---|---|---|---|---|---|
| | N | Age | Gender | SCED design | Intervention type | Setting | Implementer | SPS class | ESs | SPS-related DV domain |
| Agran et al. (2002) | 1 | 14 | 1M/0F | MB | Direct PST | School-based | Educator/para | Comprehensive SPS | 1 | Problem identification; action planning |
| Alzrayer et al. (2019) | 3 | 7.4-10.8 | 3M/0F | MB | Direct PST | School-based | Researcher | Embedded SPS component | 10 | Social requesting; response selection |
| Bock (2007a) | 1 | 12.3 | 1M/0F | MB | Direct PST | School-based | Researcher | Comprehensive SPS | 3 | Social cue interpretation; action |
| Bock (2007b) | 4 | 9.25-10.5 | 4M/0F | MB | Direct PST | School-based | Researcher | Comprehensive SPS | 12 | Social cue interpretation; action |
| Cote et al. (2014) | 3 | 9-11 | 2M/1F | MP | Direct PST | School-based | Educator/para | Comprehensive SPS | 3 | Problem identification; solution justification |
| Cox et al. (2024) | 4 | 16-19 | 4M/0F | MP | Combined | School-based | Researcher | Applied problem-solving | 4 | Financial problem correction |
| Davis et al. (2010) | 3 | 16-17 | 3M/0F | MP | Social story | School-based | Educator/para | Embedded SPS component | 3 | Social cue response |
| Dotto-Fojut et al. (2011) | 4 | 12-13 | 4M/0F | MB | Direct PST | School-based | Researcher | Applied problem-solving | 4 | Work-problem help seeking |
| Frampton & Axe (2025) | 2 | 17-18 | 1M/1F | MB | Technology-based | School-based | Researcher | Applied problem-solving | 6 | Information-seeking problem solving |
| Ingvarsson & Hollobaugh (2010) | 4 | 4-10 | 4M/0F | MP | Direct PST | School-based | Researcher | Embedded SPS component | 4 | Clarification requesting |
| Keesey-Phelan et al. (2022) | 1 | 7 | 1M/0F | MB | Combined | Clinical/community/transition | Researcher | Embedded SPS component | 3 | Self-questioning for social events |
| Lora et al. (2020) | 4 | 12-16 | 3M/1F | MB | Direct PST | School-based | Researcher | Applied problem-solving | 4 | Work-barrier resolution |
| Marckel et al. (2006) | 2 | 4-5 | 2M/0F | MB | Direct PST | Home-based | Therapist/BT | Embedded SPS component | 6 | Alternative AAC requesting |

| Study | Participant characteristics | | | Study characteristics | | | | | DV/effect-size characteristics | |
|---|---|---|---|---|---|---|---|---|---|---|
| | N | Age | Gender | SCED design | Intervention type | Setting | Implementer | SPS class | ESs | SPS-related DV domain |
| McLucas & Gonçalves (2025) | 2 | 17-20 | 2M/0F | MP | Combined | School-based | Educator/para | Embedded SPS component | 6 | Workplace adaptive responding |
| McLucas et al. (2024) | 3 | 17-23 | 3M/0F | MP | Video modeling | Clinical/community/ transition | Therapist/BT | Embedded SPS component | 8 | Workplace adaptive responding |
| Root et al. (2022) | 4 | 21 | 2M/2F | MP | Combined | School-based | Researcher | Applied problem-solving | 4 | Financial problem correction |
| Stauch & Plavnick (2020) | 2 | 16-18 | 2M/0F | MP | Video modeling | Clinical/community/ transition | Therapist/BT | Embedded SPS component | 10 | Workplace cue response |
| Suarez et al. (2022) | 3 | 10-22 | 3M/0F | MP | Combined | Home-based | Therapist/BT | Comprehensive SPS | 3 | Social conflict resolution |
| Villante et al. (2021) | 2 | 17-25 | 2M/0F | MB | Technology-based | Clinical/community/ transition | Therapist/BT | Applied problem-solving | 5 | Work-barrier resolution |
| Yakubova & Taber-Doughty (2017) | 4 | 17-20 | 4M/0F | MP | Combined | School-based | Researcher | Comprehensive SPS | 12 | Multi-step SPS routine |
| Yakubova & Zeleke (2016) | 3 | 17-18 | 3M/0F | MP | Other | Clinical/community/ transition | Researcher | Applied problem-solving | 3 | Transition-task problem solving |

***Note***. Rows are organized by study. Cases = autistic participant/case clusters included in the quantitative synthesis; ESs = DV-level effect-size estimates. SPS = social problem solving; SCED = single-case experimental design; WWC = What Works Clearinghouse; AAC = augmentative and alternative communication; PST = problem-solving training; MB = multiple baseline or multiple probe design.
